# PTSD in parents of children with severe diseases: a systematic review to face Covid-19 impact

**DOI:** 10.1186/s13052-021-00957-1

**Published:** 2021-01-14

**Authors:** Martina Corsi, Alessandro Orsini, Virginia Pedrinelli, Andrea Santangelo, Carlo Antonio Bertelloni, Niccolò Carli, Rodolfo Buselli, Diego Peroni, Pasquale Striano, Liliana Dell’Osso, Claudia Carmassi

**Affiliations:** 1 Department of Clinical and Experimental Medicine, Psychiatric Clinic, Azienda Ospedaliero-Universitaria Pisana, University of Pisa, Pisa, Italy; 2 Occupational Health Department, Azienda Ospedaliero-Universitaria Pisana, University of Pisa, Pisa, Italy; 3 Department of Clinical and Experimental Medicine, Pediatric Clinic, Azienda Ospedaliero-Universitaria Pisana, University of Pisa, Pisa, Italy; 4 Pediatric Neurology, Pediatric Department, Azienda Ospedaliero- Universitaria Pisana, University of Pisa, Pisa, Italy; 5 Department of Neurosciences, Rehabilitation, Ophthalmology, Genetics, Maternal and Child Health, University of Genoa, Genoa, Italy; 6 Pediatric Neurology and Muscular Diseases Unit, IRCCS Istituto Giannina Gaslini, Genoa, Italy

**Keywords:** PTSD, Severe diseases, Caregivers, Parents, COVID19

## Abstract

**Context:**

The literature agrees on the impact of post-traumatic stress symptoms in parents of seriously ill children but there is less clarity about the real extent and gender differences of this psychopathological risk. The recent Covid-19 outbreak highlighted new burdens for researchers on Post Traumatic Stress Disorder (PTSD) and clear evidence-based knowledge on this issue is timely needed**.**

**Objective:**

In this review, we present a synthesis of the updated evidence on PTSD rates in parents of children with severe diseases.

We also aim to try to understand if research in this field has been refined over time with the long-term intent to better face the new challenges of Covid-19 in the paediatric field.

**Data sources:**

The PubMed database was searched.

**Study selection:**

Studies were included if they assessed PTSD in parents of children diagnosed with physical illnesses.

**Data extraction:**

Of 240 studies, 4 were included.

**Results:**

Analysis of the 4 studies revealed 2 studies with PTSD rates around 20% and in line with previous best-evidence. All 4 studies tried to provide more data on fathers, however, all the studies present the lack of a control group.

**Limitations:**

The limited number of studies, which also differ widely in the methodology used.

**Conclusions:**

Methodological errors evidenced in all the 4 studies limit their reliability, making the understanding of the paediatric caregiver’s concern regarding PTSD still difficult. More sound research is needed.

## Introduction

### State of the art

Post-Traumatic Stress Disorder (PTSD) in parents of children with severe physical illnesses represents a public mental health concern that has received increasing attention over the past decade [1–6]. Indeed, PTSD is associated with substantial morbidity, diminished quality of life, high levels of medical utilization, and high economic costs, besides an important burden on the child’s care [7–12].

The most recent edition of the Diagnostic and Statistical Manual of Mental Disorders (5th ed.; DSM-5; American Psychological Association, [APA], 2013) [13] first recognized PTSD as a possible mental health sequelae to severe acute and chronic illness experiences both in patients and family members [14].

Since its first appearance in the IIIrd edition of the DSM [15], the PTSD classification, in fact, did not include the illness of a child as a traumatic event for the development of the disorder, but increasing attention has subsequently been paid to the topic in the literature, allowing its inclusion nowadays among traumatic events [13, 16].

In particular, the scientific literature addressed this phenomenon in two meta-analyses, one in 2009^17^ and one published in February 2019 [17]. The 2009 meta-analysis included sixteen studies, showing pooled PTSD prevalence rates of 19.6% in mothers, 11.6% in fathers, and 22.8% in parents. Furthermore, the pooled prevalence ratio for the four studies reporting comparison healthy groups was 4.2. Notably, in families of children with cancer, the proportion of PTSD was about 4 times higher than it was in families with healthy children [18].

These results were what stimulated the great increase of observational studies on this topic and, together with the numerous studies also carried out on other populations of traumatized subjects [19–23], contributed to determining the changes observed in the DSM-5 regarding the traumatic event characteristics. In the DSM-5 criterion A (Trauma), more attention was given to the relevance of indirect exposure to stressor events experienced by a loved one, stating that exposure to an illness that involves the actual or threatened death of a child could be a traumatic event that can lead to PTSD [13].

The DSM-5 specifies the need for the event to be violent or accidental (sudden medical catastrophe), focalizing more on the urgency and abruptness of the perceived threat rather than on its severity. This concept modifies and narrows the possibilities with respect to the previous DSM-IV-TR [16] that only required learning that one’s child had a life-threatening disease. In line with the previous DSM-IV indications, studies on this issue were mainly on cancer diseases and, to a lesser extent, on traumatic accidents. It has only been in the last few years that other diseases have emerged [24–29].

In order to advance the scientific literature on parental PTSD of the last few years, the 2019 meta-analysis could rely on around ten papers providing epidemiologically more sound results [17].

The authors first screened and highlighted 290 studies on this topic in which the most represented disease was still cancer with 159 papers, then 36 studies were on burns, 17 on diabetes, 12 heart disease, 5 epilepsy, 4 asthma, cleft lip and/or palate and phenylketonuria, 2 on HIV infection, sickle-cell disease, spina bifida, and food allergy; finally, 41 studies focussed on other infectious diseases, but at least a quarter of them were of poor quality.

Surprisingly, the results evidenced parents’ PTSD rates of 18.9%, which is perfectly in line with the results of Cabizuca et al.. Nevertheless, 14,891 subjects out of the total sample of 30,068 in Pinquart’s study was represented by parents of children with cancer, generalizing this epidemiological data at risk of bias, and contributing to the difficulty in addressing real epidemiological PTSD rates of parents of sick children in general [17].

However, it can be assumed that the prevalence rates of PTSD reported in this sub-population of traumatized individuals are higher than those reported by epidemiological studies in the general population, with prevalence rates as high as 6.8% in the United States [30] and similar rates reported in European cohorts [31, 32].

Notably, percentages of traumatic stress symptoms, while not meeting the diagnostic criteria for PTSD, were particularly evidenced in parents of children with epilepsy, diabetes, sickle cell disease, heart disease, and cancer. Contrary to expectations, parents of children with a fatal disease, such as incurable cancer or HIV infection/AIDS, did not report the highest levels of post-traumatic stress symptoms, indicating that the objective features of the traumatic event may be less important for the development of traumatic stress symptoms than the individual’s subjective experience of the event. Pinquart’s results also confirmed that the common literature recognized pre-traumatic risk factors for PTSD such as female gender and low social resources, as well the general decline of the disease over time [17].

Considering that the recent outbreak of the COVID-19 pandemic will dramatically lead to a surge of post-traumatic stress symptoms in different groups of people in the future and some evidence for this has already emerged [33], it is important to ensure the most up-to-date evidence on PTSD in all fields of research. In addition to this, even if symptoms of COVID-19 disease are usually mild in children, the high risk of infectiousness for other family members may worsen the emotional impact and the traumatic burden for parents who have to deal with with the COVID-19 infection of their children [34].

### Objectives

The present update will summarize last year’s literature published since the 2019 meta-analysis [17] on PTSD and post-traumatic stress symptoms related to having a child with severe illness. A particular focus on possible gender differences between mothers and fathers will be also addressed.

Considering the fact that over a 10-year period, the articles published on this subject showed a tenfold increase, we assumed that a significant number of articles may have been published in the last year.

The main aim in exploring these further studies is to aspire to compiling real epidemiological data on parent’s PTSD with regard to ill children. A further aim is to observe if they address the literature gaps highlighted in previous findings: the majority of the studies were on cancer, included predominantly maternal samples and a very small number had a control group.

With this aim in mind, we have selected 4 main areas of interests, in particular: parents’ PTSD rates, disease type, number of fathers compared to mothers and control groups.

Our goal is to try to understand if research in this field has been refined over time with the long-term intent to help parents better deal with the psychological challenges of having a child infected with SARS-CoV-2 (COVID-19).

## Method

### Search strategy

Since Pinquart’s meta-analysis included studies until April 2018, we conducted a Medline/PubMed search of published studies between May 2018 and April 2020.

Our digital search strategy involved the co-presence in the title of the keyword “PTSD” with at least one of the following three-word combinations: “parents” and/or “mothers” and/or “fathers”, excluding all studies dealing with other psychological aspects or concerning other mental disorders such as depression, anxiety, etc.

Other web-based databases, as well as unpublished studies, were excluded from the search. Reference sections of the identified papers were eventually checked for additional studies.

### Study selection

We included only English language papers that met the following main inclusion criteria: the study assessed PTSD in parents of children diagnosed with physical illnesses.

Eligibility was determined using an approach based on The Grading of Recommendations, Assessment, Development and Evaluation (GRADE) to assess the quality and strength of the evidence.

The selected studies were addressed using a specific “Data Collection Form (DCF)” comprising: N° of parents of children with chronic physical illness; N° of mothers: N° of fathers; type of illness; type of study; time since diagnosis; parental age; parental couple paired for one child (same index traumatic event); child’s age at the time of diagnosis; time since diagnosis; PTSD rates divided by gender.

**Fig. 1:**
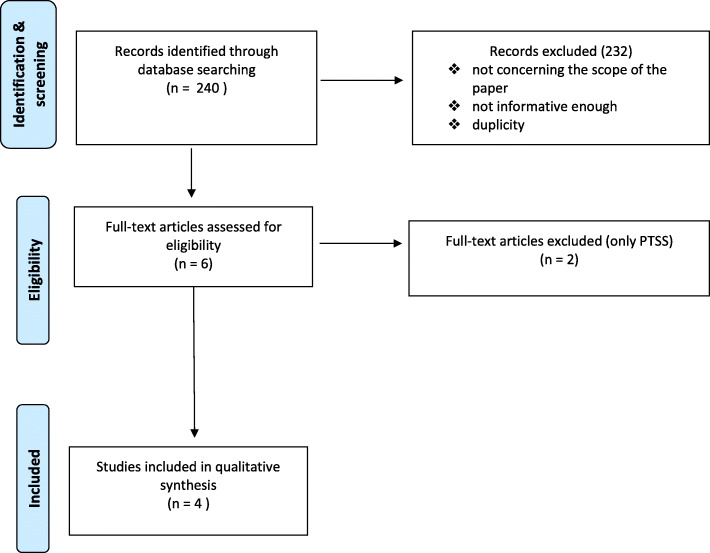
PRISMA Flow chart of study selection process

The DCF was filled in separately by two different authors (CB and VP) and checked by a third author (MC). The decisions for inclusion or exclusion are summarized in a flow chart according to the Preferred Reporting Items for Systematic reviews and Meta-Analyses (PRISMA) recommendations (Fig. 1). Due to the high degree of heterogeneity between studies, we were unable to undertake a formal meta-analysis.

## Results

### Study selection

The Medline/Pubmed search retrieved 240 results. We then excluded titles with the following features: a) not concerning the scope of the paper; b) not informative enough; c) duplicity.

Seven studies were thus selected as potentially relevant after screening of titles and abstracts. Among them, we identified 5 cross-section observational studies and 1 review. The latter was eventually discarded because it included studies described in Pinquart’s meta-analysis and the other 2 studies were ruled out because they explored Post-Traumatic Stress Symptoms (PTSS) and not PTSD diagnosis. In the end, four studies were included in this review. The corresponding authors were contacted to supply the information missing from the articles.

### Characteristics of the included studies

The characteristics of the included studies are summarized in Table 1.

**Table 1:** Key characteristics of the included studies

Study	Month, year	Type of study	Child disease	Time since diagnosis/event (years)	Total sample (N)	Mothers			Fathers			Parent couple	^Child Age (years)	PTSD (%)
				°Mean [SD] or median [IQR]		N	°(age) Mean [SD] or median [R]	*Psych fam. (%)	N	°(age) Mean [SD] or median [R]°	*Psych fam (%)		°Mean [SD] or median [R]°	
Carmassi et al.,	Feb, 2019	Cross sectional	Epilepsy	> 1 month	199	134	42.3 [27–60]	17.2%	65	45.9 [29–60]	18.5%	44	0–18	♀ 19.5% ♂ 8.1%
Werner et 'al.,	May, 2019	Cross sectional	Cardiac device	6.3 [0.4–17.9]	126	69	42 [28.0–60.0]	–	57	44 [28.0–68.0]	–	–	4.9 [4.4]	♀ 1% ♂ 0%
Lehman et al.,	Jan, 2020	Longitudinal T0 / T1 (6 months)	Stroke	♀ 0.70 [0.33–1.25] ♂ 0.58 [0.34–1.25]	81	54	36.2 [23.8–56.0]	–	27	38.4 [34.7–41.3]	–	23	1.58 [0.92–10.92]	♀ 28% ♂ 15%
Schecter et al.,	Feb, 2020	Cross sectional	NICU infants	1	91	**–**	**–**	**–**	**–**	**–**	**–**	**–**	< 1 > 1	♀ 17% ♂ 9%

° SD:Standard deviation/ R:Range/ IQR:Interquartile Range

^Child age at the time of diagnosis/*Parents’ psychiatric family history

A total of 4 studies provided data on 409 parents of children with physical illnesses. Among them, 3 papers reported ages and divided the samples by gender, with a total of 257 mothers (mean age 40.9 years) and 149 fathers (mean age 43.73 years). The physical problems of the children involved were different: 2 papers reported on neurological events, namely epilepsy and stroke, 1 on heart diseases needing implantation of a cardiac rhythm device, and 1 on infants experiencing the intensive care unit.

Two studies specifically involved younger children, with a mean age of 1.58 years [35] and around one year old [36]. The other two studies involved parents whose children presented the event at a mean age of 4.9 years [37] and children followed by paediatricians at any age (0–18 years) [38].

Two studies collected data from 6 months to one year after the diagnosis/event of the child’s disease [35, 36]; whereas one paper reported results collected at a mean time of 6.3 years after the event (Werner) [37], and another one showed data gathered generally a month after the diagnosis of the child’s disease, as required for DSM-5 PTSD diagnosis [38].

Only one study reported psychiatric familiar history in 15.6% of subjects (17.2% among mothers and 18.5% among fathers) [38].

Noteworthy, two studies showed quite homogeneous results, with rates of PTSD ranging from 17 to 19.5% among mothers and from 8.1 to 9% among fathers [36, 38], whereas the parents of patients who experienced a stroke showed a higher prevalence of PTSD (28% of mothers and 15% of fathers) [35]. Surprisingly, Werner et al. [37] reported much lower rates, since only one mother and no fathers had a diagnosis of a PTSD related to the implantation of a cardiac rhythm device in their children.

## Discussion

The present review summarizes and discusses the latest evidence regarding PTSD in parents of ill children in order to have updated data in real-time in a period in which scientific research in the field of PTSD needs to be implemented and epidemiological data are necessary.

The Covid-19 outbreak is indeed a public health emergency of international concern and poses a challenge to psychological resilience [33, 39–42]. Research data are needed to develop evidence-driven strategies to reduce adverse psychological impacts and psychiatric symptoms during the epidemic.

Two studies out of four present matching PTSD rates and in line with Pinquart’s meta-analysis [36, 38]. These results corroborate the epidemiological power of the prevalence rates elaborated by Pinquart. There is insufficient data to understand why the other two studies differ so much. One hypothesis would depend on the time between the traumatic clinical event and the parents’ PTSD assessment. Indeed, the study with the higher prevalence rates [35] has the shortest range of time since diagnosis (less than 1 year) and the study with very low rates [37] has a rather long time (almost 7 years) since diagnosis. It is possible that a period of 6.3 years offered sufficient time to recover and to develop resilience [7, 43, 44].

Nevertheless, rates of around 20% turn on a psycho-social alarm on pediatric caregivers and suggest the need for increasing multidisciplinary approaches to pediatric diseases. Social and clinical interventions should be planned by clinician teams together with psychologists and psychiatrists with sufficient expertise on various diseases to enable them to respond effectively to the peculiar needs and situations of pediatric caregivers.

### Which was the impact of previous best evidence in guiding the research in this field in the last year?

Answering this issue is fundamental to highlight useful directions to target scientific research on pediatric caregivers PTSD from this point on.

To the best of our knowledge, this is the first review on this subject that tries to analyse the impact of previous best evidence in refining subsequent research.

Methodological errors evidenced in all four studies limit their reliability, making the understanding of the pediatric caregiver’s concern regarding PTSD still difficult.

In this regard, none of the available papers included a control group which is necessary to increase the robustness of these types of studies. This observation makes us question researchers’ ability or possibility to apply evidence in practice [45]. Two different speculative reasons could be difficulty activating broad protocols that require two different study samples or a general difficulty detecting all the information on best evidence that the literature can provide.

On the other hand, all four studies were not on cancer diseases so we can, therefore, affirm that, in this case, the main meta-analysis take-home message has been received, which is that many other diseases can have a damaging effect on parents, leading them to develop real symptomatic pictures of post-traumatic stress disorder. Furthermore, the total number of fathers involved in the studies has significantly increased. This is very important in the perspective of gender medicine both in preventive and clinical terms since mothers and fathers present many differences in the response to stress.

The main limitation of this work is the analysis of a limited number of studies, which also differ widely in the methodology.

## Conclusions

More sound research is needed to deal with the psychopathological issue of PTSD in pediatric caregivers in a preventive and gender-targeted perspective.

## Data Availability

Data sharing is not applicable to this article as no datasets were generated or analysed during the current study.

## Abbreviations

PTSD: (Post-Traumatic Stress Disorder)
DSM: (Diagnostical and Statistical Manual of Mental Disorders).
APA: (American Psychiatric Association).
COVID-19: (Coronavirus disease 2019).
GRADE: (Grading of Recommendations, Assessment, Development and Evaluation).
DCF: (Data Collection Form).
PRISMA: (Preferred Reporting Items for Systematic reviews and Meta-Analyses).
PTSS: (Post-Traumatic Stress Symptoms).
